# Effects of case management in community aged care on client and carer outcomes: a systematic review of randomized trials and comparative observational studies

**DOI:** 10.1186/1472-6963-12-395

**Published:** 2012-11-14

**Authors:** Emily Chuanmei You, David Dunt, Colleen Doyle, Arthur Hsueh

**Affiliations:** 1 Centre for Health Policy, Programs and Economics (CHPPE), Melbourne School of Population Health, The University of Melbourne, Melbourne, Victoria, 3010, Australia; 2 National Ageing Research Institute, Royal Melbourne Hospital, PO Box 2127, Melbourne, Victoria, 3050, Australia; 3 Australian Catholic University, 115 Victoria Pde Fitzroy, Melbourne, Victoria, 3065, Australia

**Keywords:** Case management, Community aged care, Effects, Systematic review

## Abstract

**Background:**

Case management has been applied in community aged care to meet frail older people’s holistic needs and promote cost-effectiveness. This systematic review aims to evaluate the effects of case management in community aged care on client and carer outcomes.

**Methods:**

We searched Web of Science, Scopus, Medline, CINAHL (EBSCO) and PsycINFO (CSA) from inception to 2011 July. Inclusion criteria were: no restriction on date, English language, community-dwelling older people and/or carers, case management in community aged care, published in refereed journals, randomized control trials (RCTs) or comparative observational studies, examining client or carer outcomes. Quality of studies was assessed by using such indicators as quality control, randomization, comparability, follow-up rate, dropout, blinding assessors, and intention-to-treat analysis. Two reviewers independently screened potentially relevant studies, extracted information and assessed study quality. A narrative summary of findings were presented.

**Results:**

Ten RCTs and five comparative observational studies were identified. One RCT was rated high quality. Client outcomes included mortality (7 studies), physical or cognitive functioning (6 studies), medical conditions (2 studies), behavioral problems (2 studies) , unmet service needs (3 studies), psychological health or well-being (7 studies) , and satisfaction with care (4 studies), while carer outcomes included stress or burden (6 studies), satisfaction with care (2 studies), psychological health or well-being (5 studies), and social consequences (such as social support and relationships with clients) (2 studies). Five of the seven studies reported that case management in community aged care interventions significantly improved psychological health or well-being in the intervention group, while all the three studies consistently reported fewer unmet service needs among the intervention participants. In contrast, available studies reported mixed results regarding client physical or cognitive functioning and carer stress or burden. There was also limited evidence indicating significant effects of the interventions on the other client and carer outcomes as described above.

**Conclusions:**

Available evidence showed that case management in community aged care can improve client psychological health or well-being and unmet service needs. Future studies should investigate what specific components of case management are crucial in improving clients and their carers’ outcomes.

## Background

The Case Management Society of Australia defines case management as “a collaborative process of assessment, planning, facilitation and advocacy for options and services to meet an individual’s holistic needs through communication and available resources to promote quality cost-effective outcomes [1]. Case management is also described as a type of care delivery that has a long history of being applied in various settings, such as aged care, disability care, mental health and health care [2]. It first emerged in nursing practice in the 1800s and then was applied in social work practice in 1863 in the U.S. [3,4].

New concepts of case management addressing complex, fragmented, duplicative and uncoordinated systems arose in the 1960s in the U.S. These originated in both the mental health movement and professional social work [5]. Further associated developments in the 1970s, again in the U.S. included deinstitutionalization, the independent living movement, increased number of community-dwelling older people with complex care needs, the fragmented care delivery system, and the need for cost control [6-8]. Many developed countries, such as England, Canada and Australia, are now attempting to integrate case management approaches into their aged care systems to provide comprehensive services for community-based frail elderly people.

There is no standard definition of case management in community aged care [9]. Compared with case management in other community-based care settings (such as primary care and community mental health), distinguishing features of case management in community aged care include [10-12]:

Providing a broad span of case-managed community care and medical services for those having chronic, ongoing and complex medical conditions, and age-related disabilities, including dementia [13,14]

Providing services long-term or in intense short periods before placement in residential aged care

Involving a collaborative process with the family carer

Employing a planned approach to achieve client outcomes with cost-efficiency

Being based in the community aged care sector

With such a long history of service provision, it is not surprising that case management has been subject to considerable scrutiny over time through systematic reviews of its effectiveness. We have examined systematic reviews looking at case management that is applied in various community-based care settings and/or targets population with specific chronic diseases [15-24]. These reviews investigated a wide range of outcome domains related to care clients, carers, care organizations (e.g. service use and costs), and care delivery systems (such as care accessibility and continuity). Nevertheless, no systematic reviews to date have specifically evaluated the effects of case management in community aged care on client and carer outcomes.

As described above, case management in community aged care differs from the other types of case management in a number of ways. Evaluating the effects of case management interventions on various outcome domains concurrently may result in heterogeneity of research findings. At this stage, a more specific, targeted review of effects is warranted. Client and carer outcomes are important effect indicators, but have been attracting less research focus compared with the other outcome measures, such as service use and costs. Therefore, we conducted this systematic review with the aim summarizing the evidence for the effects of case management in community aged care on client and carer outcomes.

## Methods

Randomized control trials (RCTs) and comparative observational studies were included in this study. Due to the heterogeneity in study design, participants, interventions, outcome measures and measurement tools among studies, we conformed to the PRISMA Statement in conducting our systematic review (rather than a meta-analysis) [25]. We summarized the effects of case management in community aged care interventions based on whether the majority of studies reported significant, positive outcomes that favored the intervention group. Where the majority of available studies (in particular those with higher quality) reported that the intervention group had statistically favorable outcomes (such as greater satisfaction and better functioning status) compared with those of the control group, we reported in the results below that case management in community aged care interventions had significant effects on these outcome measures.

Study selection criteria included: no restriction on date; English language; only involving community-dwelling frail older people (suffering from age-related health problems, such as functional disabilities and dementia) and/or carers; case management interventions (excluding disease management programs that target older adults with specific chronic diseases, and specific preventive measures, such as in-home visit); care setting limited to community aged care (excluding the other community-based care settings, such as primary care, community mental health, etc.); case management as an independent intervention (rather than as a small component of a multi-faceted intervention or an integrated care delivery system/model); published in refereed journals or publications of equivalent standard; RCTs or comparative observational studies; and evaluating client and/or carer outcomes.

Based on previous studies [26-30], we focused on the following outcome variables:

Client outcomes included mortality/survival days, physical or cognitive functioning, medical conditions, psychiatric symptoms and associated behavioral problems, unmet service needs, psychological health or well-being (related to self-perceived health status, such as depression, stress, anxiety, life satisfaction etc.), and satisfaction with care.

Carer outcomes included stress or burden, psychological health or well-being, satisfaction with care, and social consequences (such as social support, and relationships with care clients—getting on well or not).

### Search strategy

We searched Web of Science, Scopus, Medline, CINAHL (EBSCO) and PsycINFO (CSA) from inception to July 2011. We also used Google Scholar to identify studies that did not appear in these databases. Table 1 shows the Medical subject heading (MeSH) search terms and keywords. The Medline search strategy was applied to the other databases where Title/keywords/abstract was available. Reference lists of the retrieved articles and those systematic reviews as mentioned above were also checked to find potential articles.

**Table 1:** Summary of Medline Search strategy

1	(case management ), key term
2	exp case management/or exp managed care program/
3	(care management),key term
4	exp nursing care/or exp managed care program/or exp self care
5	1 or 2 or 3 or 4
6	((care coordination) or (channeled care) or (care advocacy) or (care integration) or (integrated care) or (key worker) or (service broker) or (community matron) or linkage or brokerage), key term
7	5 or 6
8	(community care), key term
9	exp community networks/
10	(respite care) , key term
11	exp respite care/
12	(home care) , key term
13	exp foster home care/or exp home care services/or exp home nursing/ or home care agencies/ or home health aides/or exp patient-centered care/or exp delivery of health care, integrated
14	(long-term care ) , key term
15	exp long-term care/or exp insurance, long-term care
16	(home health) , key term
17	exp home care services/or home health aides/
18	(social service*), key term
19	exp social welfare/ or social work/
20	8 or 9 or 10 or 11 or 12 or 13 or 14 or 15 or 16 or 17 or 18 or 19
21	(community aged care) or (day care)or(home assistance) or (home help) or(in-home) or (community-based care) or (home-based care) or (aged care) or (senior care) or (elder* care) or (social care) , key term
22	20 or 21
23	7 and 22
24	limit 23 to ((English language; people aged 65 or over, or 80 or over; and human)
25	23 and 24

### Data extraction and synthesis

We downloaded all searched studies into EndNote 4.0 software. The first author (EY) independently screened titles and abstracts of all originally searched articles (3704 in total). If doubt existed, the second author (DD) reviewed the abstracts. Following this step, EY and DD reviewed abstracts and/or full texts of all potential, relevant articles (141 in total). EY reviewed full texts of most articles at least once. DD reviewed abstracts and where necessary full texts of these articles. After this process, EY and DD compared their results.

EY and DD independently extracted information on the characteristics (country of origin, sample size, participants, length of follow-up and intervention details), and client and carer outcomes of the studies.

Divergence regarding data extraction and synthesis was addressed through discussion between EY and DD.

### Quality assessment

EY and DD also independently assessed the quality of included studies by using a checklist (see Table 2) that was informed by previous systematic reviews, the PRISMA Statement and the Cochrane Handbook for Systematic Reviews of Interventions [23-25,31]. 

**Table 2:** Quality assessment of the studies

**Author**	**Overall quality**	**Methodology quality**						
		**Quality control**	**Randomization**	**Comparability**	**Follow-up rates**	**Dropout**	**Blinding assessor**	**Analysis**
Lam (2010)	High quality	+	+	+	90.2%	+	+	+
Yordi (1997)	Moderate quality	+	+	+	34.0%	+	?	?
Newcomer (1999)	Low quality	+	+	+	36.0%	?	?	?
Applebaum (1988)	Low quality	?	?	?	?	?	?	?
Rabiner (1995)	Low quality	-	?	?	100%	+	?	?
Lowenstein (2000)	Moderate quality	+	+	+	95.0%	+	?	?
Eloniemi-Sulkava (2001)	Moderate quality	+	+	+	52.0%	+	?	?
Applebaum (2002)	Moderate quality	+	+	+	82.9%	+	?	?
Shapiro (2002)	Moderate quality	+	+	+	50.0%	+	-	?
Challis (1985)	Low quality	+	-	+	47.0%	+	?	?
Marek (2006)	Low quality	+	-	+	71.8%	+	-	?
Specht (2009)	Low quality	+	-	-	34.9%	+	-	?
Onder (2007)	Low quality	+	-	-	71.6%	+	-	?
Miller (1985)	Low quality	-	-	-	?	?	?	?
Marshall, (1999)	Low quality	+	?	-	91.5%	+	-	?

Note:

Quality control: Whether case management interventions were described clearly;

Group comparability: Whether baseline characteristics of the intervention and control groups were similar;

Follow -up rate: Whether the percentage of follow-up was complete;

Dropouts: Whether dropouts were clearly enumerated and/or compared with those completed cases at baseline;

Blinding assessor: Whether assessment was conducted by independent interviewers blinded to group or objective outcomes;

Analysis: Whether intention-to-treat analysis was applied

“+” means “yes”, “-” means “no”, “?” means “no details”

High quality studies: providing full information on all the seven items (follow-up rate being over 90% was regarded as “full information”); moderate quality studies: providing information on at least four items; low quality studies: providing information on fewer than four items.

Written informed consent was obtained from the patient for publication of this report and any accompanying images.

## Results

**Figure 1:**
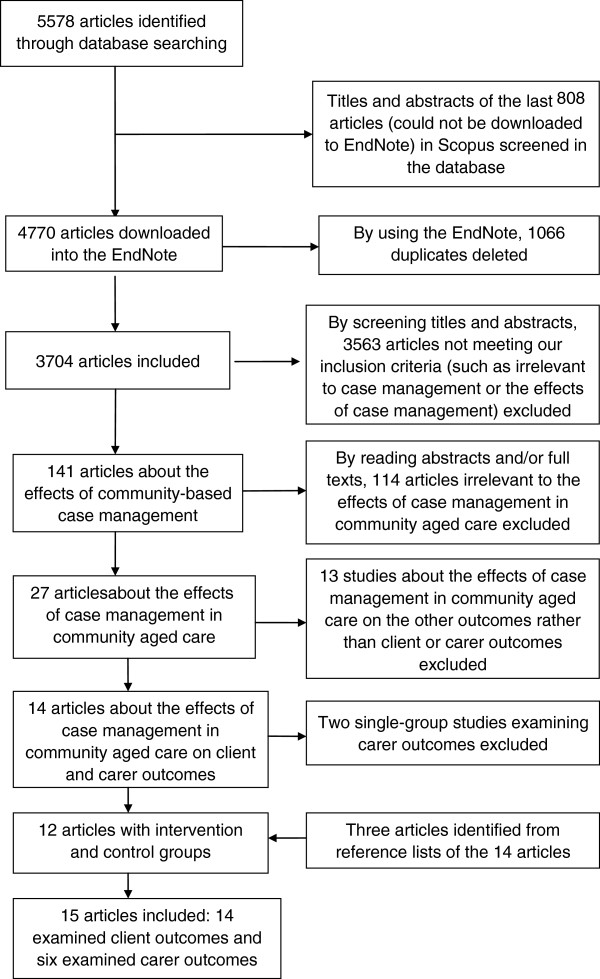
Study selection process.

The study selection process yielded 3704 articles in total (see Figure 1). After the exclusion process, 141 full-text articles were retrieved and reviewed, with 15 studies finally included in this review. The 15 selected studies involving 13 case management in community aged care programs were summarized in Table 3[30,32-45]. Ten studies were from the USA and one each from England, Hongkong, Finland, Italy and Israel. Only four studies were published after 2005[30,41-43]. 

**Table 3:** Characteristics of studies included in the systematic review

**Author, year & location**	**Study design, study sample (mean age of older pepole X where applicable), sample size (N) & intervention length**	**Case management in community aged care interventions**	**Measurement instrument**	**Client outcomes**	**Carer outcomes**
Yordi (1997) USA	RCT Older people with dementia Intervention/control: X=78.3/X=78.3; N= 2,707/N=2,547 3 years	Needs assessment, assisting carers arranging services, activating care plan & care quality monitoring. Intervention: smaller caseload (n=30) & higher monthly benefits ($430- $699 per client), control: larger caseload (n=100) & lower monthly benefits ($290- $489 per client) (the Alzheimer’s disease demonstration program).	Functional status: measured by a version of the Katz ADL & Lawton and Brody’s IADL scale at six-month intervals	Robust effect on reducing unmet needs with ADL/IADL tasks over time Significantly fewer unmet service needs in the intervention group during different follow-up periods	
Newcomer (1999) USA	RCT Carers of older people with dementia Intervention/control: X=63/X=63; N=2,731/N=2,576 3 years	See [32].	Burden: measured by an adapted scale developed by Zarit, Reever and Bach-Peterson Depression: measured by the short-form Geriatric Depression Scale		Burden: no significant intervention-control group differences during 6-, 12- or 36-month follow-up Depression: no significant intervention-control group differences during 12- or 36-month follow-up
Applebaum (1988) USA	RCT Older people (eligible for nursing home admission) & carers Intervention/control: N=1,861/N= 2,013 18 months	Needs assessment, care planning, service arrangement, monitoring, care plan modification & reassessment. Intervention: case managers having control over pooled funds; control: adopting a brokerage model (the Channeling Demonstration program). See [46]	Mortality rates Functioning: measured by an ADL five- item (eating, transfer, toileting, dressing, bathing & continence) scale, an IADL seven-item (housekeeping, meal preparation, shopping, transportation, taking medicine, financial management & telephone use) scale, and number of days restricted to bed Client social/psychological well-being: overall life satisfaction, morale, attitude towards to aging, social interactions, self-perceived health& overall contentment index Carer social/psychological well-being: life satisfaction & relationship with clients Carer stressors: perceived emotional/physical/financial strain & number of stressful behavior problems	No significant intervention-control group differences in mortality rates during different follow-up periods (first six months, 7–12 months & 12–18 months) Significantly higher life satisfaction, fewer number of unmet service needs & fewer number of ADL disabilities in the intervention group during different follow-up periods No significant intervention-control group differences in the number of IADL disabilities & number of days restricted to bed during different follow-up periods	Satisfaction with service arrangements improved significantly over time No significant intervention-control group differences in satisfaction, social well-being/psychological & stressor measures during different follow-up periods
Rabiner (1995) USA	RCT People aged 65 and over Six-month analysis: N=2,237; 12-month analysis: N=1,726 (no details about the sample size of each group) 1 year	See [46]	Satisfaction: measured by extent of confidence (not confident, somewhat confident and very confident) in care at six- & 12-month follow-up	No significant intervention-control group difference in satisfaction during six-month follow-up Significantly greater satisfaction in the intervention group during 12-month follow-up	
Lowenstein (2000) Israel	RCT People aged 69 and older (most of whom suffering from physical, cognitive and mental diseases) & carers Intervention/control: N=30/N=30 1 year	Referral, intake, needs assessment, care plan activation, care linkage, care plan modification, informal care support, monitoring, reassessment, evaluation & discharge arrangement.	Outcomes: reported by social workers through individual/group interviews at the end of the study	Significant improvements in community participation, satisfaction with services, consumer choice & unmet needs in the intervention group.	Significant improvements in satisfaction with services & burden in the intervention group.
Eloniemi-Sulkava (2001) Finland	RCT People aged 65 and older with dementia Intervention/control: X=78.8/X=80.1; N=53/N= 47 2 years	Advocacy, counseling, annual training, follow-up calls, in-home visits, care arrangement & 24-hour services.	Year 1 &2 death rates	No significant intervention-control group differences in death rates during different follow-up periods	
Applebaum (2002) USA	RCT Older people with disabilities Intervention/control: X=78.2/X=79.5; N=154/N= 154 18 months	Preventive activities (assessment & consumer training) & intervention activities (communication with physicians & hospital discharge planning).	Death rates & mean number of survival days by six, 12 and 18 months Functioning status: measured by average number of ADL disabilities (bathing, dressing, transfer from bed to chair, getting to the toilet, eating& inside mobility), average number of IADL disabilities & cognitive disorder (having Alzheimer/Dementia/ other cognitive or not) at different time points (baseline, six- & 12-month follow-up) Overall health status (range 0–16), overall health and service satisfaction (range 0–20): measured at different time points	No significant intervention-control group differences in death rates, mean number of survival days, service satisfaction, health status & physical functioning during different follow-up periods	
Shapiro (2002) USA	RCT Older adults on waiting list to receive social care Intervention/control: X=77.8/X=76.9;N=40/N=65 18 months	Geriatric assessment subsequent service provision, care planning, care coordination & contacts per 3 months.	Nursing home admission/death rate by 18 months Quality of life (including depression, social satisfaction, mastery & life satisfaction): measured at three-month intervals	The intervention group was significantly less likely to die or be institutionalized Significantly better quality of life in the intervention group during different follow-up periods	
Miller (1985) USA	Quasi-experimental study Low-income elderly medical people at risk of institutionalization Intervention/control: X=79/X=76.5 & N=1068/N=1495 in community group. X=77/X=76.1 & N=983/N=848 in hospital group. X=80.9/X=81.8 & N=261/N=196 in nursing home group. X=77.9/X=90 & N=607/N=28 in target group 2 years	A team of case managers providing multi-dimensional assessment, care planning, service arrangement, follow-up and reassessment	Number of days of life saved within one year and two years respectively	Significantly increased longevity among participants in the intervention group by 3.97 days in 1981, and 7.25 days 1982 Interventions were most effective for the frailest clients	
Marshall, (1999) USA	RCT People aged 65 and over Intervention/control: X=81/X=82;N=159/N=160 2 years	Screening, selection, assessment, treatment plan, service arrangement within and outside the program & periodic reassessment	Functional status: measured by ADLs (bathing, eating, transferring, toileting &dressing rated from 1 to 3. higher score meant higher dependency level) and IADLs (needing telephoning, transportation, walking, & medication services or not) at baseline, year 1 & year 2 Perceived health status: measured on a scale of 1 to 4 defined as excellent, good, fair, or poor respectively at baseline, year 1 & year 2 Satisfaction with care: measured on a scale of 1 to 5 from very satisfied to very dissatisfied at baseline, year 1 & year 2	During one-year follow-up: significantly improved perceived health status in the intervention group; control group was more satisfied with care; no significant intervention-control group differences in functional status measures During two-year study period: significantly better functional status in the intervention group Time effects: the intervention group did not experience significant changes in functional status or perceived health status during the whole study period compared with the control group	
Lam (2010) Hongkong, China	RCT People aged 65 years old or above with mild dementia Intervention/control: X=78.6/X=78.2;N=59/N=43 18 months One year (with 4 months’ intervention)	Assessment and advice, home-based program on cognitive stimulation, liaising with other care professionals to ensure clients and carers’ participation in community activities	Carer stress: measured by Zarit Carer Burden Interview (ZBI) (22 items, specifically including perceived health, psychological well-being, financial impact, social life, and carer and client relationships) at three-month intervals (below is the same) Carer psychological health: measured by general health questionnaire (GHQ) Carer subjective quality of life: measured by personal well-being Index for Adults (PWI-As) Client cognitive status: measured by Mini Mental State Examination (CMMSE) Client psychiatric symptoms and behavioral disturbance: measured by The Neuropsychiatric Inventory (NPI) Client personal well-being: measured by the Personal Well-Being Index-Intellectual Disability (PWI-ID) Client depression: measured by Cornell Scale for Depression in Dementia (CSDD)	Significant improvement in client depression in the intervention group during 4-month follow up No significant intervention-control group differences in client cognitive status, client psychiatric symptoms and behavioral disturbances, or personal well-being during any study periods	No significant intervention-control group differences in carer stress, psychological health, or subjective quality of life during any study periods
Challis (1985) England	Quasi-experiment Older people eligible for nursing home admission & carers Intervention/ control: N=74/N=74 3 years	Referrals, assessment, care planning, monitoring & case closure.	Death rates: measured at one-year intervals Client quality of life (morale, depressed mood, anxiety, loneliness & felt capacity to cope) & Carer outcomes (level of subjective burden; extent of strain; mental health difficulties; difficulties in social life, household routine, employment& financial issues): measured at the end of the study	No significant intervention-control group difference in death rate by 24 months; significantly lower death rate in the intervention group by 36 months. Significantly better quality of life (except anxiety) in the intervention group	Significantly lower level of burden in the intervention group No significant intervention-control group differences in the other carer outcomes
Marek (2006b) USA	Quasi-experiment The frail elderly aged 64 and older Intervention/ control: N=55/N=30 1 year	Needs assessment, care plan reviewing, monitoring & hospital care coordination	Functioning status: measured by ADL (bed mobility, transfers, locomotion, eating & toileting. Each item scored from 0–4) score, cognitive performance scale (range 0–6) at different time points (baseline, six- & 12-month follow-up) Depression rating scale (range 0–2 for observed frequency of each of the seven mood indicators): measured at different time points Frequency of health outcomes measured at different time points: including incontinence (range 0–2), pain (range 0–3), dyspnea (range 0–4)& ability of medication management (range 0–1)	More death cases (6 vs.2) by 12 months in the intervention group. No significant intervention-control group differences in all client outcomes during six months Significantly better ADL performance, and less pain & dysnea in the intervention group during 12 months.	
Specht (2009) USA	Quasi-experiment Older people with dementia & carer Intervention/control: X=82 .4/X=78.5;N=167/N=82 1.5 months	Needs identification and assessment, care plan development, home visits, monthly phone contacts, quarterly face-to-face contacts, periodic reassessment & care system coordination.	Outcomes of 3–9 months & 9–15 months were assessed Client functioning: measured by MMSE (range 1–30), GDS (range 1–7), functional Assessment II, Groff, R.L (range 1–3), modified IADL/ADL’s from Lawton and Brody (range 1–5) Client behaviors: measured by a rating checklist (Garrity and Klein) Client & carer health status: measured by SF-36 Carer well-being, stressors & endurance potential:measured by NOC (Moorehead et al.)	Significant decline in ADL abilities in the intervention group from baseline to each follow-up No significant intervention-control group differences in ADL disabilities, MMSE, GDS & behavior rating index during different follow-up periods.	Significantly lower stress, and better endurance potential & well-being in the intervention group during different follow-up periods
Onder (2007) Italy	Retrospective cohort Frail elderly people Intervention/control: X=82 .1/X=82.5;N=1,184/N= 2,108 1 year	Initial assessment, monitoring, additional care provision, care plan design and implementation, care arrangement & care coordination.	1-year mortality	No significant intervention-control group difference in 1-year mortality	

As shown in Table 3, there were ten RCTs and five comparative observational studies in this area. Studies varied in their designs, the nature of case management interventions, their specific outcome variables and measurement tools. In these 15 studies, follow-up periods ranged from one to three years, and sample size varied from 60 to 8095.

All the studies were based on demonstration/pilot programs that targeted community-residing elderly people with some age-related health problems (such as functional disabilities and dementia) and/or their carers.

As reported by these studies, case management in community aged care interventions generally included assessment, care planning, care plan implementation, care coordination, monitoring, and reassessment. Where programs targeted people with dementia and their family carers, specific intervention components could include education and counseling services, carer training, medical treatment and medication management, crisis interventions, client empowerment, and client advocacy. The two programs in the USA—the Channeling Demonstration and Evaluation program [34,35] and the Alzheimer’s Disease Demonstration program[32,33], increased financial benefits and allowed case managers to make independent decisions on resource allocation. Comparators were usual care for the control group and case management interventions for the experimental group.

According to Table 2, there was one high-quality RCT study (providing information on the seven items—follow-up rate reaching over 90% was regarded as “full information”) [30], five moderate-quality studies (providing information on at least four items), and nine low-quality studies (providing information on fewer than four items).

### Intervention effects on the client

14 studies reported client outcomes, including mortality/survival days (7 studies), physical or cognitive functioning (6 studies), medical conditions (2 studies), psychiatric symptoms and associated behavioral problems (2 studies), unmet service needs (3 studies), psychological health or well-being (7 studies), and satisfaction with care (4 studies).

While mortality, physical or cognitive functioning, medical conditions, psychiatric symptoms and associated behavioral problems, and unmet service needs are objective measures, psychological health or well-being and satisfaction with care are more subjective indictors.

#### Mortality/survival days

Of the seven studies examining mortality/survival days, two reported a significant effect of case management in community aged care interventions (hereafter case management interventions) on reducing client mortality or lengthening survival days.

One moderate-quality RCT reported that participants in the intervention group were significantly less likely to die or be admitted to residential care during the 18-month study period [39], while another low-quality quasi-experimental study found that the intervention group increased longevity significantly during the first and second years [44]. The other five studies all (including one low-quality RCT, one moderate-quality RCT, two low-quality quasi-experimental studies and one low-quality retrospective cohort study) reported no significant intervention-control group differences in client mortality of their different study periods [34,38,40,41,43].

#### Physical or cognitive functioning

Six studies investigated clients’ physical functional status and/or cognitive functioning, but reported inconsistent results across different measures during different study periods. For example: Some studies found that case management interventions could significantly improve the performance of ADL-related tasks (but not IADL-related tasks or cognitive status measured by GDS/MMSE) among the intervention participants; others showed that the interventions only had significant effects in the long-term rather than in the short-term.

Three low-quality studies provided some evidence that case management interventions had a long-term effect on ADL measures. One low-quality RCT found that participants in the intervention group had significantly fewer number of ADL disabilities during the six months, 7–12 months, and 12–18 months of follow-up respectively [34]. This study, however, found no significant intervention-control group differences in the number of IADL disabilities or the number of days restricted to bed during these study periods [34]. Another low-quality RCT found no significant intervention-control group difference in client functional status after one year, but reported significantly better functional status among intervention participants after two years [45]. A third low-quality quasi-experimental study reported that participants in the intervention group had significantly better ADL performance after 12 months, but indicated no significant intervention-control group differences in physical or cognitive status after six months [41].

Three other studies, however, indicated no clear effect of case management interventions on ADL or cognitive functioning. One moderate-quality RCT reported no significant intervention-control group differences in the number of ADL or IADL disabilities during six-month and 12-month follow-up periods respectively [38]. One high-quality RCT reported no significant intervention-control group differences in client cognitive status during any study periods [30]. And one low-quality quasi-experimental study showed no significant intervention-control group differences in ADL/IADL index, minimum-mental state examination (MMSE), or GDS during the 3–9 months and 9–15 months of follow-up [42].

#### Medical conditions

Of the two studies examining client medical conditions, one low-quality quasi-experimental study reported less pain and dyspnoea among participants in the intervention group during the 12-month follow-up period [41], while the other moderate-quality RCT showed no significant intervention-control group differences in client health status during six-month, 12-month and 18-month study periods [38].

#### Psychiatric symptoms and associated behavioral problems

Two studies (including one high-quality RCT and one low-quality quasi-experimental study) examining these outcomes did not find significant intervention-control group differences during their different study periods [30,42].

#### Unmet service needs

Improving unmet service needs as an outcome showed more evidence for successful application of case management in community aged care. Three studies, including two moderate-quality RCTs and one low-quality RCT, consistently reported that case management interventions had significant effects on improving clients’ unmet service needs [32,34,36].

#### Psychological health or well-being

Psychological health and wellbeing had more evidence for a good outcome of case management interventions for older clients. Of the seven studies evaluating this outcome, five (including two low-quality RCTs, two moderate-quality RCTs and one low-quality quasi-experimental study) reported that case management interventions had significant effects on improving intervention participants’ psychological health or well-being across different measures, such as self-perceived life satisfaction, morale, depression, mastery, and personal health status [34,36,39,40,45].

One high-quality RCT reported no significant intervention-control group differences in clients’ personal well-being, but showed significant improvement in depression among participants in the intervention group during different study periods [30].

The remaining one low-quality quasi-experimental study revealed no significant intervention-control group difference in client depression after six months [41].

#### Satisfaction with care

Four studies examined this outcome, with three reporting no significant effects of case management interventions on improving client satisfaction with care services.

One moderate-quality study found that the intervention group improved satisfaction with service provision during one-year study period [36]. One low-quality RCT documented significantly higher satisfaction among participants in the intervention group during the 12-month study period, but found no significant intervention-control group difference during the six months of follow-up [35].

In contrast, one moderate-quality RCT demonstrated no significant intervention-control group differences at any study periods [38], while another low-quality RCT found that the control group had significantly higher satisfaction with care during one-year study period [45].

### Intervention effects on the carer

Six studies reported carer outcomes, including carers’ stress or burden (6 studies), satisfaction with care (2 studies), psychological health or well-being (including perceived health conditions, life satisfaction, psychological distress, depression etc.) (5 studies), and social consequences (such as social support and relationships with clients) (2 studies). All these outcome measures, as we observe, are related to carers’ subjective feelings.

#### Stress or burden

The six studies examining this outcome reported variable effects of case management interventions on carer stress or burden.

One moderate-quality RCT reported significant improvement in the burden of carers in the intervention group during the one-year study period [36]. Another two low-quality quasi-experimental studies showed significantly lower level of stress or burden among carers in the intervention group during their different study periods [40,42]. In contrast, two low-quality RCTs [33,34] and one high-quality RCT [30] reported no significant intervention-control group differences in carer burden or stress during their different study periods.

#### Satisfaction with care

Of the two studies reporting carer satisfaction, one moderate-quality RCT demonstrated significantly higher satisfaction among carers in the intervention group over the one-year study period [36], while the other low-quality RCT found no significant intervention-control group differences during any study periods [34].

#### Psychological health or well-being

Of the five studies investigating this outcome, only one low-quality quasi-experimental study reported that participants in the intervention group had significantly better well-being during different follow-up periods [42]. Conversely, four studies (including one high-quality RCT, two low-quality RCTs, and one low-quality quasi-experimental study) did not find significant intervention-control group differences during their different study periods [30,33,34,40].

#### Social consequences

Two studies (including one low-quality RCT and one low-quality quasi-experimental study) reported no significant intervention-control group differences in carer social consequences, such as carers’ social life and carers’ relationships with their clients [34,40].

## Discussion

Community care is increasingly the preferred mode of care for older people to avoid residential care. While consumer-directed care for this section of the population is gaining popularity, a large proportion of older people will continue to use case managers to assist them in negotiating their care needs [15]. This review provided largely consistent evidence that case management interventions improve older clients’ psychological health or well-being and also deliver significant improvements in unmet service needs. Clear effects of the interventions on other client outcomes and carer outcomes are not so evident, with mixed evidence for the other outcome variables reviewed here. We found that studies reported inconsistent results regarding client physical or cognitive functioning and carer stress or burden. There was also limited evidence supporting that case management in community aged care interventions improve client length of survival, health conditions, behavioral problems or satisfaction with care, as well as carer satisfaction with care, psychological health or well-being and social consequences.

There are a number of limitations to these conclusions. First, the number of studies involved was small. While we identified ten RCTs, only one RCT was rated high quality according to our quality criteria [30]. One RCT did not describe the study design [34], requiring us to obtain this information elsewhere [46]. Information about blinding assessors and intention-to-treat analysis was commonly missing in most studies. Other methodological limitations of many studies include small sample size, and lack of information on sample size calculation or strategies of controlling confounding factors.

Assessment, care planning, care plan implementation, client advocacy, monitoring, review, and case closure were reported as the core case management functions in community aged care setting, but many studies did not provide full information about the intensity, breadth and duration of each function. This poses a challenge to attribute different client and carer outcomes to specific intervention components.

As with most systematic reviews, we found that variations in the nature, content and individual components of case management interventions or functions, as well as absence of information on the intervention implementation make it challenging to compare the results among different studies [8,47].

The choice of outcome measures that are appropriate or valid is critical to a fair evaluation of the effects of case management in community aged interventions. The studies reviewed here used a large number of outcome measures with little justification of their appropriateness or robustness [48]. Some studies also reported that the instruments used for outcome assessment were inconsistent over time or among different research participants, again leading to challenges in drawing evidence-based conclusions [33,38].

Another prominent issue is that the studies reviewed here used diverse instruments or methods to measure different client and carer outcomes. For instance, clients’ physical or cognitive functioning was measured by the number of ADL/IADL limitations, ADL/IADL score, MMSE, and/or GDS; unmet service needs were reported by clients, carers or care professionals.

An overarching question for further research is the ‘dose-response’ relationship between case management interventions and client and carer outcomes. Some researchers claim that more focused but less intensive or comprehensive case management in community aged care interventions can be effective [49,50]. Other researchers accentuate that the intensity of the interventions should be strong enough—at least different from that of the usual care—to achieve desired outcomes [32,33].

Participants in some case management in community aged care programs were not chosen with specific inclusion criteria in mind; for example, individuals with low-risk of nursing home admission were unexpectedly enrolled by some programs targeted at reducing nursing home admission. This may partly explain why the case management interventions did not achieve some desired outcomes [34]. This finding lends support to previous research, indicating that problems in recruiting suitable participants hamper many programs in demonstrating their success [5,51-53].

Although we did not find systematic reviews specifically assessing the effects of case management in community aged care interventions on client and carer outcomes, our findings, to some degree, were consistent with previous related systematic reviews that examined the effects of case management interventions on various outcomes.

First, previous reviews reported that the effects of case management interventions on many client outcomes were inconclusive. For example, one review revealed that most included studies found no significant intervention-control group differences in client satisfaction, physical functioning, mortality, or quality of life [54]. Regardless of different care settings, study populations, and interventions previous reviews and our study focused on, case management interventions cannot improve all client outcomes. Our findings here suggest that case management interventions alone might not reverse or significantly improve some health conditions in the frail elderly.

Second, previous reviews concluded that case management interventions have moderate or no significant effects on carer burden and depression [24,28,29,55,56]. One reason for this finding is that it might be difficult to improve carer outcomes in reality, since caregiving always leads to carers experiencing high levels of stress, burden and other negative consequences; or the finding could be attributed to measurement difficulties. Furthermore, many case management interventions include no or only moderate intervention components for carers themselves. This should be addressed in designing new case management programs in future, if carer outcomes are one of the target goals.

In general, this study answers the review question: “What are the effects of case management in community aged care interventions on carer and client outcomes?” The evidence from this review may enlighten policy makers to design appropriate case management interventions and reasonable intervention goals in the area of community aged care in future. Moreover, it may advise care professionals to focus on the areas where the interventions have significant effects, so as to make appropriate decisions on resource allocation in their practice.

### Limitations

This systematic review is limited by the methodological shortcomings in most studies, e.g. nine studies were rated as low-quality studies, while five were classified as moderate-quality studies. Other limitations are as follows:

First, we did not review studies that compared different types of case management models or focus on case management as a component of multifaceted interventions. But valuable information can be obtained from multifaceted interventions, only if case management components can be separated from the whole intervention [57].

Second, we did not search studies published in non-English Journals or grey literature. We have noted that most included studies were from the United States.

Finally, because of limited resources, we did not use a pre-specified protocol to guide the conduct of our systematic review. Since review of the effects of case management/case management in community aged care on various outcomes is ongoing [58], this issue should be addressed in the future.

## Conclusions

Available evidence in this review showed that case management in community aged care interventions can improve client psychological health or well-being and unmet service needs. In contrast, the effects of the interventions on client mortality, functional status, medical conditions, behavioral problems and satisfaction with care services, as well as carer outcomes as noted by this review are less conclusive.

Future studies should investigate what specific components of case management are crucial in achieving improved outcomes for the client and their carer. In addition, undertaking evaluation studies by employing rigorous study designs are warranted.

## Abbreviations

(RCTs): Randomized control trials; (ADL): Activities of daily living; (IADL): Instrumental activities of daily living; (MMSE): Minimum-mental state examination; (GDS): Global dementia scale.

## Competing interests

There are no competing interests.

## Authors’ contributions

DD&EY had equal contributions to conceptualizing this paper, as well as screening literature, reviewing studies for inclusion, extracting data, and writing and critically reviewing the paper. CD conceptualized and critically reviewed the paper. All the authors interpreted data, read and approved the final manuscript.

## Authors’ information

EY is a PhD candidate in the Centre for Health Policy, Programs and Economics (CHPPE), School of Population Health, The University of Melbourne. Professor DD is the founding director of the Centre for Health Policy, Programs and Economics (CHPPE), School of Population Health, The University of Melbourne. He is a public health specialist and medically-trained epidemiologist, with expertise in health program evaluation and health service research. Dr. CD is a professor of Aged Care at Australian Catholic University/Catholic Homes and the director of Service Development & Evaluation Division in the National Ageing Research Institute (NARI). She has expertise in aged care research and health program evaluation. Dr. AH is a senior lecturer & health economist in the Centre for Health Policy, Programs and Economics (CHPPE), School of Population Health, The University of Melbourne.

## Pre-publication history

The pre-publication history for this paper can be accessed here:

http://www.biomedcentral.com/1472-6963/12/395/prepub
